# Artificial Microglia Nanoplatform Loaded With Anti‐RGMa in Acoustic/Magnetic Feld for Recanalization and Neuroprotection in Acute Ischemic Stroke

**DOI:** 10.1002/advs.202410529

**Published:** 2024-10-30

**Authors:** Ruiqi Cheng, Xiaoqin Luo, Xiaohui Wu, Zijie Wang, Ziqun Chen, Shaoru Zhang, Hongmei Xiao, Jiaju Zhong, Rongrong Zhang, Yang Cao, Xinyue Qin

**Affiliations:** ^1^ Department of Neurology The First Affiliated Hospital of Chongqing Medical University Chongqing 400016 China; ^2^ Chongqing Key Laboratory of Ultrasound Molecular Imaging Institute of Ultrasound Imaging Ultrasound Department of the Second Affiliated Hospital of Chongqing Medical University Chongqing 400010 China; ^3^ Department of Rehabilitation Medicine Yongchuan Hospital of Chongqing Medical University Chongqing 402160 China

**Keywords:** ischemic stroke, microglia membrane, nanoplatform, neuroprotection, recanalization

## Abstract

Ischemic stroke is a leading cause of death and disability worldwide, and the main goals of stroke treatment are to destroy the thrombus to recanalize blood vessels and protect tissue from ischemia/reperfusion injury. However, current recanalization therapies have serious limitations and there are few neuroprotection methods. Hence, an artificial nanoplatform loaded with anti‐Repulsive Guidance Molecule a monoclonal antibody (anti‐RGMa) and coated with microglia membrane (MiCM) is reported for stroke treatment, namely MiCM@PLGA/anti‐RGMa/Fe_3_O_4_@PFH (MiCM‐NPs). Tail vein injection of MiCM‐NPs targeted the ischemia‐damaged endothelial cells because of the MiCM, then superparamagnetic iron oxide (Fe_3_O_4_) and anti‐RGMa are released after external low‐intensity focused ultrasound (LIFU) exposure. The thrombus is destroyed by LIFU‐induced “liquid‐to‐gas” phase transition and cavitation of perfluorohexane (PFH) as well as Fe_3_O_4_ movements induced by an external magnetic field. Anti‐RGMa protected the ischemic region from ischemia/reperfusion injury. The nanoplatform enabled visualization of the thrombus by ultrasound/photoacoustic imaging when the clot is in an extracranial artery. Importantly, in vivo animal studies revealed good safety for MiCM‐NPs treatment. In conclusion, this nanoplatform shows promise as an ischemic stroke treatment strategy combining targeted delivery, recanalization, and neuroprotection.

## Graphical abstract

This research reports an artificial nanoplatform namely MiCM@PLGA/anti‐RGMa/Fe_3_O_4_@PFH: PFH as a core, loaded with anti‐RGMa and Fe_3_O_4_, coated with microglia membrane (MiCM) for stroke treatment. The nanoplatform targets the ischemia‐damaged endothelial cells of thrombus site because of MiCM, and Fe_3_O_4_ and anti‐RGMa are released after LIFU exposure. The thrombus is destroyed by PFH and Fe_3_O_4_. Anti‐RGMa protects the ischemic region.

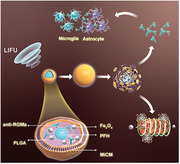

## 1 Introduction

Ischemic stroke is a leading cause of death and disability worldwide, thus imposing a severe global health burden. The key points of stroke treatment involve destroying the thrombus to recanalize blood vessels and protecting tissue from ischemia/reperfusion injury. Currently, the widely used recanalization treatments for acute ischemic stroke are intravenous administration of tissue plasminogen activator (tPA) and mechanical thrombectomy (MT).^[^ ^1^, ^2^ ^]^ However, tPA has a low recanalization rate ranging from 4% to 33%, so the chance for neurological recovery is unfavorable.^[^ ^3^, ^4^ ^]^ MT has a higher recanalization rate, but it also has a higher risk of intraoperative complications (e.g., vascular injury, inguinal hematoma, entrapment), mainly related to the site of thrombectomy.^[^ ^3^ ^]^ Furthermore, ischemia/reperfusion injury occurs after recanalization, which is associated with poor outcomes in patients with successful recanalization.^[^ ^5^ ^]^ Triggered by ischemia/reperfusion, ischemia/reperfusion injury is a complicated pathophysiological process that includes blood‐brain barrier disruption, inflammatory response, and reactive astrogliosis. In recent years, emerging preclinical data have shown that neuroprotection treatment adjunctive to reperfusion therapy reduces infarct volume and improves neurological function by protecting the ischemic region from reperfusion injury.^[^ ^6^ ^]^ Since neuroprotection drugs as an adjunct to recanalization therapy could provide tangible clinical benefits, it is desirable to develop an integrated targeted‐ischemic stroke treatment strategy that combines thrombus destruction with an effective neuroprotection drug.

With the emergence of nanotechnology, a series of mechanical clot dissolution strategies based on smart responsive nanomaterials have been developed for arterial and venous thrombosis in preclinical studies, such as ultrasound and magnetic responsive platforms.^[^ ^7^ ^]^ Ultrasound‐responsive microbubbles are highlighted as a potential theranostic system for both ultrasound visualization of thrombus and clot destruction by mechanical stress. Low‐intensity focused ultrasound (LIFU), a non‐invasive ultrasound technique that reduces conventional ultrasound‐induced energy damage and precisely focuses ultrasound on the target site, has been demonstrated to destabilize and destroy clots by triggering the phase‐change materials encapsulated in nanoparticles in animal models of abdominal aortic thrombosis^[^ ^8^ ^]^ and coronary thromboembolism.^[^ ^9^ ^]^ However, to the best of our knowledge, there are few studies focused on LIFU‐triggered thrombus destruction for stroke treatment. LIFU could target cerebral structures through the intact skull.^[^ ^10^ ^]^ The challenge is that prolonged LIFU exposure may produce thermal damage in brain tissue, but brief LIFU exposure may not destroy the thrombus completely. Hence, a LIFU and magnetic combination‐responsive nanoplatform is proposed in the current study. Magnetic‐responsive materials can move and rotate in a targeted manner under the manipulation of a magnetic field. In recent years, nanoparticles loaded with magnetic‐responsive materials, such as superparamagnetic iron oxide (Fe_3_O_4_), have shown great potential in both thrombus visualization and destruction with the concomitant use of a thrombolytic drug.^[^ ^11^ ^]^ It has been reported that Fe_3_O_4_‐microrods encapsulated with tPA improve tPA‐induced thrombolysis by disrupting the clot network when applied with an external rotating magnetic field.^[^ ^12^ ^]^ Hence, we propose a LIFU and magnetic combination‐responsive nanoplatform to achieve safe and effective recanalization of blood vessels. This nanoplatform destroys the thrombus through the “liquid‐to‐gas” phase transition of perfluorohexane (PFH) and cavitation of PFH induced by LIFU as well as the rotation of Fe_3_O_4_ triggered by an external magnetic field produced by Helmholtz coils. Moreover, the phase transition of PFH can significantly enhance the visualization of ultrasound images of B‐mode and contrast‐enhanced ultrasound (CEUS),^[^ ^13^ ^]^ and Fe_3_O_4_ could serve as a photoacoustic imaging contrast agent.^[^ ^14^ ^]^ These imaging modalities contribute to the diagnosis of thrombosis, specifically when the clot is located in an extracranial artery (e.g., cervical segment thrombosis).

Repulsive guidance molecule‐a (RGMa) is a glycosylphosphatidylinositol‐anchored membrane protein belonging to the repulsive guidance molecule (RGM) family. RGMa is involved in many physiological and pathological processes of the central nervous system.^[^ ^15^, ^16^ ^]^ Our previous research demonstrated that RGMa expression is upregulated in multiple types of cells in ischemic areas after cerebral ischemia/reperfusion, including neurons, astrocytes, and endothelial cells.^[^ ^17^, ^18^ ^]^ RGMa promotes blood‐brain barrier (BBB) disruption, reactive astrogliosis,^[^ ^17^ ^]^ and inflammation.^[^ ^19^ ^]^ RGMa inhibition reduces infarct volume and promotes neurological function recovery.^[^ ^17^, ^18^, ^19^ ^]^ RGMa may be a key target for neuroprotection after cerebral ischemia/reperfusion. Anti‐RGMa monoclonal antibody (anti‐RGMa) has been shown to reduce disease severity and promote neurological recovery in multiple disease animal models, including neuromyelitis optica^[^ ^20^ ^]^ and spinal cord injury.^[^ ^21^ ^]^ It should be noted that RGMa has been reported to promote resolution and tissue repair in a murine acute peritonitis model.^[^ ^22^, ^23^ ^]^ To avoid potential side effects and enhance therapeutic efficacy, it would be best to target anti‐RGMa to the ischemic region. Therefore, we propose the strategy of loading anti‐RGMa in the targeted nanoparticle so that it can protect ischemic areas from ischemia/reperfusion injury after recanalization in ischemic stroke treatment.

Targeted delivery is important for recanalization and neuroprotection following stroke. Cell membrane coating is a promising biomimetic targeting technology. Cell membrane‐coated nanoparticles excel at interacting with biological substrates, thus enabling them to effectively navigate complex biological environments, avoid immune clearance, and accumulate at disease sites.^[^ ^24^ ^]^ Microglia are the resident immune cells and the first line of defense against injury in the central nervous system.^[^ ^25^ ^]^ Microglia membrane (MiCM)‐coated nanoparticles have been reported to reduce phagocytosis of macrophages and simultaneously enhance targeting to glioblastoma multiforme.^[^ ^26^ ^]^ To our knowledge, few previous studies have used microglia membrane as a coating material to target ischemic regions after stroke. Almost immediately after the onset of stroke, the BBB is disrupted, and microglia rapidly migrate towards ischemic regions and contact the damaged endothelial cells of BBB in the occluded vessel area.^[^ ^25^, ^27^, ^28^ ^]^ Thus, a microglia membrane coating may enable nanoparticles (NPs) to access the thrombus by targeting the ischemia‐damaged endothelial cells of the BBB.

**Scheme 1:**
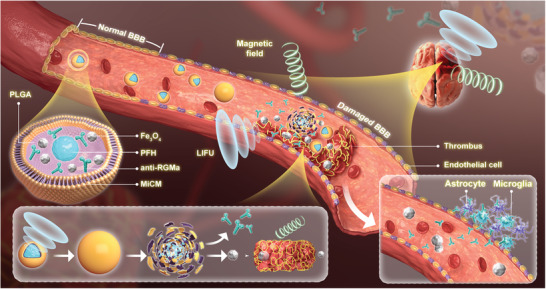
Schematic illustration of the structure and process of MiCM‐NPs in stroke. The MiCM‐NP is composed of PFH as the core, PLGA loaded with Fe_3_O_4_ and anti‐RGMa as the shell, and a microglia membrane. After delivery, the MiCM‐NPs target the ischemia‐damaged endothelial cells of the BBB due to microglia membrane coating. Then the nanoparticles are activated by LIFU to release Fe_3_O_4_ and anti‐RGMa. The thrombus is destroyed by LIFU‐induced “liquid‐to‐gas” phase transition and cavitation of PFH as well as magnetic field‐controlled Fe_3_O_4_ movements and rotation. Then anti‐RGMa protects ischemic regions from ischemia/reperfusion injury.

In the present study, we report a MiCM‐coated and LIFU/magnetic responsive nanoparticle loaded with anti‐RGMa (that is, MiCM@PLGA/anti‐RGMa/Fe_3_O_4_@PFH nanoparticle, MiCM‐NP) for targeted delivery, recanalization, and neuroprotection after stroke. The MiCM‐NP is composed of PFH as the core, poly lactic‐co‐glycolic acid (PLGA) loaded with Fe_3_O_4_ and anti‐RGMa as the shell, and a microglia membrane. **Scheme** **1** shows the structure of the MiCM‐NP and illustrates the process of targeted delivery, recanalization, and neuroprotection. After delivery, the MiCM‐NPs target the ischemia‐damaged endothelial cells of the BBB because of their microglia membrane coating. Then the nanoparticles are activated by LIFU to release Fe_3_O_4_ and anti‐RGMa. The thrombus is destroyed by LIFU‐induced “liquid‐to‐gas” phase transition and cavitation of PFH as well as magnetic field‐controlled Fe_3_O_4_ movements and rotation. Anti‐RGMa protects ischemic regions from ischemia/reperfusion injury. In addition, the MiCM‐NPs enable visualization of the thrombus by ultrasound/photoacoustic imaging when the clot is in an extracranial artery. Our data suggest that MiCM‐NPs recanalized the occluded artery, reduced the infarct volume, and promoted neurologic function through targeted delivery, recanalization, and neuroprotection. This nanoplatform may be a promising approach to stroke treatment.

## 2 Results and Discussion

### 2.1 Characterization of MiCM‐NPs

**Scheme 2:**
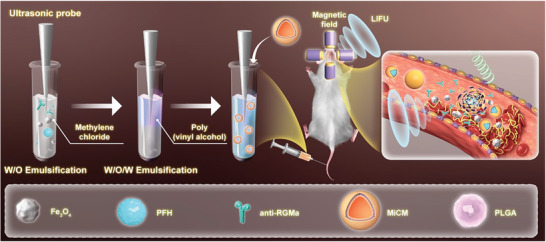
Illustration of MiCM‐NPs preparation. Fe_3_O_4_, anti‐RGMa, PFH and MiCM were added into PLGA solution in sequence to synthesize MiCM‐NPs by ultrasonic probe. The nanoparticle was injected into eMCAO mice via tail vein for recanalization and neuroprotection.

**Figure 1:**
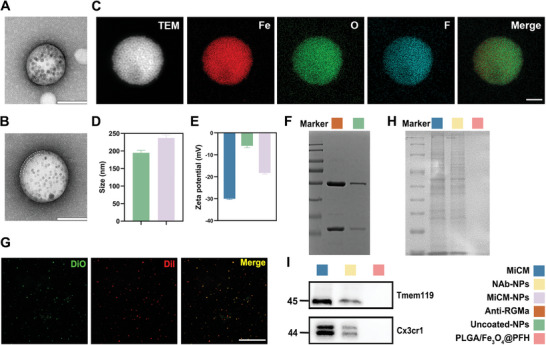
Characterization of MiCM‐NPs. Transmission electron microscopic (TEM) images of uncoated‐NP A) and MiCM‐NP B). Scale bar, 100 nm. C) High‐resolution dark‐field TEM image of MiCM‐NP and the corresponding elemental mapping of Fe, O, and F. Scale bar, 100 nm. D) Size of uncoated‐NP and MiCM‐NP. E) Zeta potentials of microglia membrane, uncoated‐NP, and MiCM‐NP. F) SDS‐PAGE analysis of proteins of anti‐RGMa and uncoated‐NPs. G) Colocalizations of uncoated‐NPs (labeled by green DiO) and MiCM (labeled by red Dil). Scale bar, 100 µm. H) SDS‐PAGE analysis of proteins of MiCM, NAb‐NPs, and PLGA/Fe_3_O_4_@PFH. I) Western‐blot analysis of MiCM, NAb‐NPs, and PLGA/Fe_3_O_4_@PFH for its surface marker protein (Tmem119 and Cx3cr1).

Anti‐RGMa, Fe_3_O_4_ and PFH were dissolved sequentially in an organic phase containing the polymer PLGA to fabricate PLGA/anti‐RGMa/Fe_3_O_4_@PFH NPs, and then coated the MiCM via sonication method (**Scheme** **2**). Transmission electron microscopy (TEM) of the MiCM‐NPs and PLGA/anti‐RGMa/Fe_3_O_4_@PFH NPs (uncoated‐NPs) showed their spherical morphology and revealed that dark Fe_3_O_4_ particles were incorporated into the PLGA shells. MiCM was not coated on uncoated‐NPs (**Figure** **1**A), but on MiCM‐NPs (Figure 1B; Figure S1, Supporting Information). The distribution of Fe_3_O_4_ within the NPs is clearly displayed by high‐resolution TEM images. The elemental mapping images of ferrum (Fe) and fluorine (F) demonstrated that the Fe_3_O_4_ and PFH were loaded in the NPs (Figure 1C). The encapsulation efficiency and drug loading of anti‐RGMa in MiCM‐NPs were 42.340 ± 2.872% and 16.004 ± 1.086%, respectively. The average size of uncoated‐NPs was 194.767 ± 7.385 nm, while that of MiCM‐NPs was larger at 236.967± 8.756 nm (Figure 1D). The zeta potentials of MiCM, uncoated‐NPs, and MiCM‐NPs were measured as −30.03 ± 0.289 mV, −5.93 ± 0.826 mV, and −18.27 ± 0.503 mV, respectively (Figure 1E). Sodium dodecyl sulfate polyacrylamide gel electrophoresis (SDS‐PAGE) was used to detect anti‐RGMa in uncoated‐NPs. The results confirmed that anti‐RGMa was encapsulated in the nanoparticles (Figure 1F). Then uncoated‐NPs were labeled by green DiO dye, and MiCM was labeled by red DiI dye. Uncoated‐NPs and MiCM colocalization was observed under an inverted fluorescence microscope (Figure 1G). Inherent properties relied on the specific proteins and ligands expressed on the surface of MiCM. We identified the surface marker proteins by SDS‐PAGE and western blot analysis. SDS‐PAGE results (Figure 1H) indicated that the total protein profile remained on the MiCM@PLGA/Fe_3_O_4_@PFH NPs (NAb‐NPs) after probe sonication, whereas PLGA/Fe_3_O_4_@PFH NPs showed no presence of proteins. Western blot analysis (Figure 1I) showed that MiCM special marker proteins Tmem119 and Cx3cr1 were expressed in NAb‐NPs, which further confirmed that the MiCM was infused.

### 2.2 Magnetic Field Establishment and Magnetic Verification

A past study reported that nanoparticles loaded with Fe_3_O_4_ had the ability to disrupt the network of the clot, as Fe_3_O_4_ was superparamagnetic and could be manipulated to move by magnetic fields.^[^ ^29^ ^]^ Therefore, we conducted a series of in vitro experiments to explore the ability of MiCM‐NPs to destroy the thrombus.

**Figure 2:**
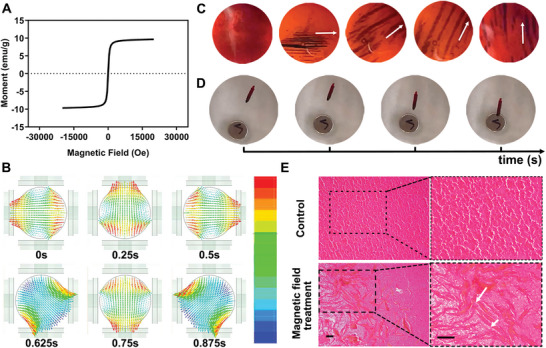
Magnetic field establishment and Fe_3_O_4_ locomotion. A) Hysteresis loop test of MiCM‐NPs. B) Direction change of rotating magnetic field at different times at frequency of 1 Hz. C) Fe_3_O_4_ locomotion in the magnetic device. D) The locomotion of the clot exposed to rotating magnetic field. E) Representative H&E staining images of blood clots received magnetic field‐controlled Fe_3_O_4_ treatment in vitro. White arrowhead indicates small channels in the clot after receiving magnetic field‐controlled Fe_3_O_4_ treatment. Scale bar, 50 µm.

We first observed the color of the nanoparticles to determine whether Fe_3_O_4_ was encapsulated in the NPs. The NPs loaded with Fe_3_O_4_ (MiCM‐NPs) were brown, whereas the NPs without Fe_3_O_4_ (MiCM@PLGA/anti‐RGMa@PFH) were white (Figure S2, Supporting Information). Subsequently, we analyzed the magnetic property of MiCM‐NPs. No residual magnetization was observed from the hysteresis loop measurement for MiCM‐NPs, indicating that the NPs were superparamagnetic (**Figure** **2**A). The rotating Fe_3_O_4_ was expected to destroy blood clots under a rotating magnetic field, so we validated the effect of Fe_3_O_4_ locomotion in the fluid environment caused by the blockage. To simulate the change in the fluid environment caused by the blockage, in vitro evaluations were conducted using a microflow pump to make the liquid flow with a velocity of 3 mL min^−1^. In the presence of magnets (Movie S1, Supporting Information), a substantial proportion of the magnetic material accumulated around the magnets, illustrating Fe_3_O_4_ can locomote in the fluid environment. Then we established an electromagnetic actuation system composed of two pairs of Helmholtz coils to explore the locomotion behavior of Fe_3_O_4_. A change in the magnetic field was simulated to verify that the direction of Fe_3_O_4_ could be controlled. The changes in the direction of the rotating magnetic field were plotted for different time intervals, and one revolution was simulated within 1 s (Figure 2B), which was consistent with the theory of applying an orthogonal current and demonstrating the homogeneity of the magnetic field, and it was crucial for the precise manipulation of Fe_3_O_4_ locomotion. Different trajectories of the electromagnetic actuation system were observed (Figure 2C). Moreover, we investigated the thrombolytic effect of Fe_3_O_4_ motion in vitro. The clot was positioned in the magnetic field (Figure S3, Supporting Information). After 60 min, the clot was removed and washed in phosphate buffered saline (PBS), at which point it exhibited magnetic adhesion (Figure 2D, Movie S2, Supporting Information). The thrombolytic effect was also verified by examination of hematoxylin and eosin (H&E) stained slices. After receiving magnetic field controlled‐Fe_3_O_4_ treatment, small channels were found in the clot (Figure 2E). The results showed that magnetic field controlled‐Fe_3_O_4_ could drill holes in the thrombus, thus promoting disruption of the thrombus.

### 2.3 Multimodal Imaging of MiCM‐NPs in Vivo and in Vitro

**Figure 3:**
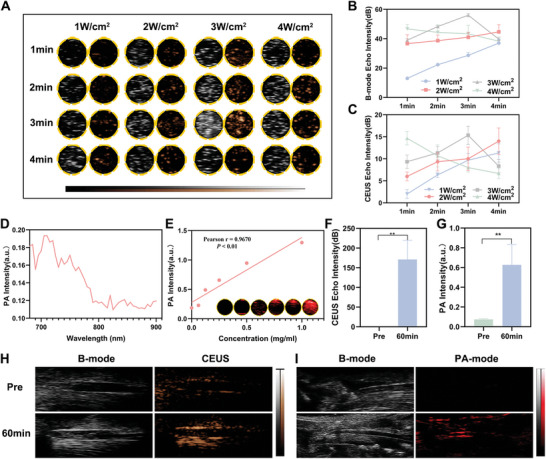
Multimodal imaging of MiCM‐NPs in vivo and in vitro. A) B‐mode and CEUS images of MiCM‐NPs after LIFU irradiation with different power. B) Echo intensity values of B‐mode after LIFU irradiation with different power (n = 3). C) Echo intensity values of CEUS after LIFU irradiation with different power (n = 3). D) PA intensity of MiCM‐NPs at the wavelength range of 680−900 nm. E) PA imaging and intensity of the MiCM‐NPs at different concentrations in vitro. F) Echo intensity values in CEUS of the two groups before and after LIFU irradiation (3 W cm^−2^, 3 min) (n = 3). G) PA intensity values in PA‐mode before and after injection (n = 3). H) B‐mode and CEUS images of left common carotid artery embolization model of SD rats received uncoated‐NPs injection pre‐LIFU irradiation and post‐irradiation (3 W cm^−2^, 3 min) (n = 3). I) B‐mode and PA‐mode images of left common carotid artery embolization model of SD rats received uncoated‐NPs injection pre‐LIFU irradiation and post‐irradiation (n = 3). Data show the mean ± SD. Data comparisons are made using student's paired t‐test; **p < 0.01. PA, Photoacoustic.

Visualizing the thrombus is vital for diagnosing and treating thrombotic diseases. The MiCM‐NPs can undergo liquid‐gas phase change under temperature or LIFU irradiation, which enhances CEUS signal. The suitable parameters of temperature and LIFU irradiation for the visualization of our NPs were assessed. In the range of 40–55 °C (measured at intervals of 5 °C) in vitro, the echo intensity in both B‐mode and CEUS of the MiCM‐NPs gradually increased (Figure S4, Supporting Information). However, this temperature exceeds the optimum temperature for living animals, which may lead to thermal damage. Subsequently, we evaluated the LIFU‐stimulated phase‐transition property of MiCM‐NPs, as shown in **Figure** **3**A. Quantitative analysis of the echo intensity in both B‐mode and CEUS of the MiCM‐NPs showed a peak at 3 W cm^−2^ for 3 min (Figure 3B,C), which suggested that the PFH encapsulated in the NPs could allow the NPs to act as an ultrasound contrast imaging agent. Moreover, photoacoustic imaging is a non‐invasive and radiation‐free imaging method that has rapidly developed in recent years. By acoustically detecting photons absorbed by tissue, photoacoustic imaging breaks through the resolution limitations of pure optical imaging, achieving the rich contrast of optical imaging as well as the high‐resolution of ultrasound imaging in living tissues.^[^ ^30^ ^]^ In the present study, we investigated the ability of photoacoustic imaging to offer visualization of MiCM‐NPs since Fe_3_O_4_ has been acknowledged as a promising photoacoustic imaging contrast agent.^[^ ^8^ ^]^ Based on full spectrum scanning from 680 to 900 nm (interval = 5 nm) in photoacoustic imaging, we observed that the wavelength of 705 nm was the optimal wavelength for MiCM‐NPs (Figure 3D). Then the photoacoustic imaging performance of the MiCM‐NPs was assessed in vitro at 705 nm. As depicted in Figure 3E, photoacoustic intensity of MiCM‐NPs was positively correlated with their concentration.

Then we assessed the in vivo visualization ability of uncoated‐NPs on a left common carotid artery (LCCA) embolization rat model. 60 min after uncoated‐NPs injection and LIFU exposure, the ultrasound echo signal of the thrombus was enhanced (Figure 3F,H). 60 min after administration and LIFU exposure, there was a notable photoacoustic signal in the LCCA embolization rat model (Figure 3I). Quantitative analysis of the photoacoustic signal intensity of the two groups showed that the photoacoustic intensity of the administration and LIFU exposure group was 8.47 times higher than the treatments before (Figure 3G), demonstrating the desirable performance of MiCM‐NPs as photoacoustic imaging contrast agents.

Collectively, the above results indicate that MiCM‐NPs hold promise as a multimodal imaging agent that could facilitate the integration of ultrasound and photoacoustic imaging into a single nanoplatform. These imaging modalities may contribute to the diagnosis of thrombosis, specifically when the clot is located in an extracranial artery (e.g., cervical segment thrombosis).

### 2.4 Targeting Ability of MiCM‐NPs in Vivo and in Vitro

The effective targeting behavior of NPs is crucial for precise drug delivery. Coating a biomimetic membrane onto the surface of nanocarriers has been reported to confer them with natural properties from the original cell membrane, such as reduced immunogenicity, prolonged half‐life, and remarkable competency in recognizing antigens for enhanced targeting.^[^ ^31^ ^]^ Macrophage membrane (MaCM)‐coated nanoparticles have been reported to have an ability of targeting to the cerebral ischemic regions via macrophage‐membrane protein‐mediated recognition with cell adhesion molecules that are overexpressed on the damaged vascular endothelium.^[^ ^32^ ^]^ Microglia is the only macrophage population in the central nervous system. After ischemic stroke, microglia adjacent to thrombus become activated and highly express metalloproteins, reactive oxygen species (ROS), vascular endothelial growth factor (VEGF), and pro‐inflammatory cytokines. This provokes a rapid disintegration of blood vessels, which subsequently leads to leakage of serum components. These serum proteins attract and activate distant microglia, which migrates towards the endothelial cells of thrombus‐induced disrupted artery, which is adjacent to thrombus. The migration ability of activated microglia is mediated mainly by Cx3cr1 that expressed on microglia surface.^[^ ^25^, ^33^ ^]^ It should be noted that our results showed that Cx3cr1 is expressed on our nanoparticle (Figure 1H). Furthermore, the migration of microglia to the damaged vascular endothelium is much more quickly than macrophage after stroke.^[^ ^34^ ^]^ Thus, we speculated that coating our NPs in microglia membrane may promote their ability to efficiently target the ischemia‐damaged endothelial cells of the BBB around the thrombus area after stroke. The targeting ability of microglia membrane‐camouflaged NPs is superior to that of macrophage membrane‐coated NPs.

**Figure 4:**
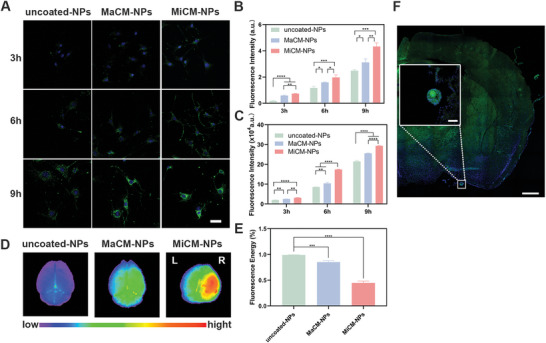
Targeting ability of MiCM‐NPs in vivo and in vitro. Representative images A) and quantification B) of bEnd.3 cells (DAPI, blue) after a 6 h‐OGD treatment incubated with DiO‐labeled (green) PLGA/anti‐RGMa/Fe_3_O_4_@PFH (uncoated‐NPs), MaCM@PLGA/anti‐RGMa/Fe_3_O_4_@PFH (MaCM‐NPs), and MiCM@PLGA/anti‐RGMa/Fe_3_O_4_@PFH (MiCM‐NPs) in different time (3 h, 6 h, 9 h). Scale bar, 50 µm (n = 3). Flow cytometric analysis C) of DiO fluorescence intensities of bEnd.3 cells with a 6 h‐OGD treatment in various experimental groups (n = 3). Ex vivo fluorescence images D) and quantification E) of the brains collected from different treatments at 6 h post‐intravenous nanoparticle injection (n = 3). F) Fluorescence images of embolus in the middle cerebral artery of eMCAO mice after injection of DiO‐labelled MiCM‐NPs (green). Scale bar, 500 µm. Insets showed higher magnification image of the clotted middle cerebral artery. Scale bar, 100 µm. The cells were stained with DAPI. Data show the mean ± SD. Data comparisons are made using one‐way analysis of variance (ANOVA) with Tukey's post hoc test and student's paired t‐test; *p < 0.05, **p < 0.01, ***p < 0.001, ****p < 0.0001.

To test our hypothesis, the targeting ability of MiCM‐NPs was evaluated and compared with macrophage membrane‐coated nanoparticles (that is, MaCM@PLGA/anti‐RGMa/Fe_3_O_4_@PFH, MaCM‐NPs) in vitro and in vivo. The macrophage membrane was successfully coated onto the surface of uncoated‐NPs (Figure S5, Supporting Information). The targeting ability of the NPs to ischemia‐damaged endothelial cells was firstly evaluated in vitro. As shown in **Figure** **4**A, when DiO‐labeled uncoated‐NPs, MaCM‐NPs, and MiCM‐NPs were incubated with mouse brain microvascular endothelial cells (bEnd.3 cell) after 6 h of oxygen‐glucose deprivationOGD) for 3, 6, and 9 h, inverted fluorescent microscopy (Figure 4B) and flow cytometry (FCM) analysis (Figure 4C) demonstrated that the targeted groups (MaCM‐NPs and MiCM‐NPs) exhibited an increasing amount of green fluorescence in bEnd.3 cells compared with the uncoated‐NPs group. And more MiCM‐NPs accumulated around the blue‐stained nuclei of cells compared with the MaCM‐NPs. The results demonstrated that microglia cell membrane endowed NPs with better cell‐targeting ability, allowing them to be taken up by bEnd.3 cells. Furthermore, the targeting capability of the NPs was assessed in vivo. The DiR‐labeled NPs were injected into the embolic middle cerebral artery occlusion (eMCAO) mice via the tail vein. Fluorescence imaging showed that the targeted groups (MaCM‐NPs and MiCM‐NPs) accumulated prominently in the damaged area. Compared with the MaCM‐NPs, the accumulation of the MiCM‐NPs in the damaged area was significantly increased (Figure 4D,E). The accumulation of MiCM‐NPs in the vital organs was evaluated. There was no obvious difference in the accumulation of uncoated‐NPs and MiCM‐NPs in heart, lung, liver, spleen, and kidney (Figure S6, Supporting Information). Additionally, by injecting DiO‐labeled MiCM‐NPs into eMCAO mice, we found that the nanoparticles accumulated in the embolus area in the right middle cerebral artery of eMCAO mice (Figure 4F). Moreover, the circulation half‐lives of the NPs were calculated since prolonged circulation time may provide more opportunities for NPs to enter the damaged site. After tail vein injection of the DiR‐labeled uncoated‐NPs and MiCM‐NPs, we collected plasma at 1, 2, 6, 12, 24, and 48 h and detected the fluorescence intensity (Figure S7, Supporting Information). Compared with the uncoated‐NPs, MiCM‐NPs had a longer blood half‐life and reduced clearance. The results above indicated that MiCM‐NPs could target the thrombus site in brain and evade immune elimination.

### 2.5 Recanalization of MiCM‐NPs

**Figure 5:**
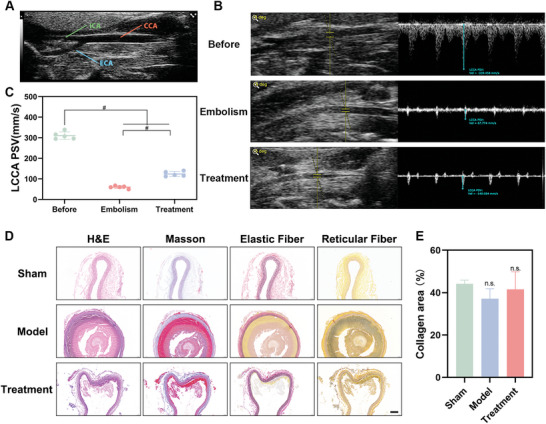
Recanalization effect of MiCM‐NPs. A) B‐mode ultrasound image of the normal structure of left cervical blood vessels. B) The pulsed wave Doppler (PW Doppler) and peak systolic velocity (PSV) of the left common carotid artery (LCCA PSV) in different groups. C) Quantification of LCCA PSV (n = 5). D) Histology of cross section of left common carotid artery in SD rats (H&E Staining, Masson Staining, Elastic Fiber Staining, Reticular Fiber Staining). Scale, 100 µm. E) Quantification of collagen content in Masson Staining (n = 3). Data show the mean ± SD. Data comparisons are made using one‐way analysis of variance (ANOVA) with Tukey's post hoc test and student's paired t‐test; ^#^ *p* < 0.0001, n.s. no significance.

The therapeutic efficacy of MiCM‐NPs was assessed in vivo. First, we evaluated the vascular recanalization efficacy of uncoated‐NPs on rat LCCA embolization (**Figure** **5**A). Uncoated‐NPs were injected into the rats through the tail vein 60 min after LCCA embolization, and the spectrum was immediately monitored by pulsed wave Doppler. After embolism, the spectrum of the distal end of the LCCA changed into a high resistance artery flow pattern with a low peak systolic velocity (PSV) at 59.897 ± 6.690 mm s^−1^, suggesting the blockage of LCCA. With the treatment of uncoated‐NPs, the PSV increased significantly, which indicated the high recanalization rate and patency of the LCCA in the therapy group (LCCA PSV = 124.000 ± 12.266 mm s^−1^) (Figure 5B,C). Because of the high intraoperative complication rate of MT,^[^ ^3^ ^]^ we were interested in detecting whether LIFU irradiation could lead to vascular wall injury. We harvested the LCCA after treatment. The treated areas of the arteries did not show obvious signs of damage which included medium elastic plate deformation, excessive collagen accumulation, detachment of endothelial cells, local necrosis, thermal damage, or inflammatory reaction (Figure 5D,E). Taken together, these results showed that MiCM‐NPs recanalized clotted vessels effectively and safely.

### 2.6 Neuroprotection Effect of MiCM‐NPs

**Figure 6:**
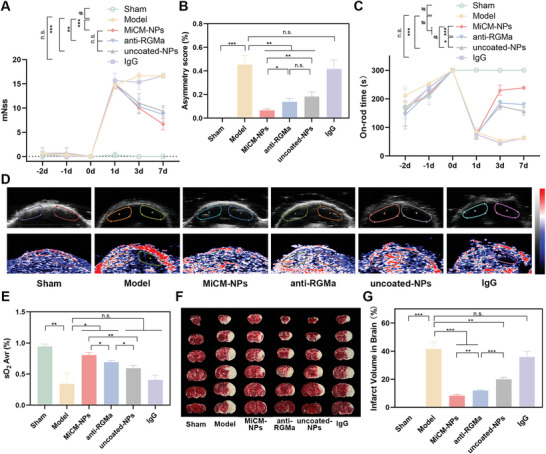
Neuroprotection effect of MiCM‐NPs. A) Modified neurological severity score was performed at 2 days before as well as 1, 3, and 7 days after eMCAO in rats (n = 3). B) Cylinder test is performed at 7 days after eMCAO in rats (n = 3). C) Rotarod test was performed at 2 days before as well as 1, 3, and 7 days after eMCAO in rats (n = 3). D) Ultrasound and PA functional images of brain sO_2_ of eMCAO model mice received different treatment. E) Statistical results of sO_2_ mapping in ischemic areas in different groups (n = 3). F) Infarct volume was determined by TTC staining. G) Quantitative calculation of the infarct volume (n = 3). Data show the mean ± SD. Data comparisons area made using one‐way analysis of variance (ANOVA) with Tukey's post hoc test and student's paired t‐test; *p < 0.05, **p < 0.01, ***p < 0.001, ^#^ *p* < 0.0001. n.s. no significance. MiCM‐NPs, MiCM@PLGA/anti‐RGMa/Fe_3_O_4_@PFH; uncoated‐NPs, PLGA/anti‐RGMa/Fe_3_O_4_@PFH.

Our previous studies confirmed that knockdown of RGMa reduced infarct volume and promoted functional recovery after cerebral ischemia/reperfusion injury.^[^ ^17^ ^]^ RGMa may be a key target for neuroprotection after cerebral ischemia/reperfusion. Therefore, we encapsulate anti‐RGMa into our nanoparticles for neuroprotection. FITC‐labeled anti‐RGMa was used to determine the encapsulation efficiency and drug loading of MiCM‐NPs. The drug loading efficiency was 16.004 ± 1.086% and release rate of anti‐RGMa at 3 W cm^−1^ for 3 min was 43.393 ± 3.842% (Figure S8, Supporting Information). Then, we evaluated the neuroprotection efficacy of nanoparticles on the eMCAO mice. The eMCAO mice were subjected to random division into five groups, each receiving a distinct drug injection. The groups included Model (saline), MiCM‐NPs, anti‐RGMa, uncoated‐NPs, and IgG. The behavioral tests of the mice were performed 2 days before as well as 1, 3, and 7 days following eMCAO. Modified neurological severity scores (mNss) were established to evaluate the functional recovery of eMCAO mice, including motor function, sensory acuity, balance, and reflex. A cylinder test was used to evaluate limb‐use asymmetry. A rotarod test indicated the swiftest and best motor function recovery. MiCM‐NPs, uncoated‐NPs, and anti‐RGMa treatment significantly attenuated the eMCAO‐induced increase in mNss score (**Figure** **6**A) and cylinder test score (Figure 6B) and the decrease in time on the rod in rotarod test (Figure 6C) after eMCAO. In the cylinder test and rotarod test, MiCM‐NPs group displayed less limb‐use asymmetry and better motor function recovery than uncoated‐NPs group and anti‐RGMa group. As for mNss, though there was no significant difference among MiCM‐NPs, uncoated‐NPs, and anti‐RGMa group, there was a trend that MiCM‐NPs group displayed lower mNss score. These results showed that MiCM‐NPs promoted neurofunctional recovery. In addition, photoacoustic imaging is a technique that is capable of excellent functional imaging of hemodynamics, though limited studies have used it for monitoring blood oxygen function after stroke.^[^ ^30^ ^]^ In this study, we evaluated oxygen saturation (sO_2_) levels in the brains of the eMCAO mice 7 days after stroke based on photoacoustic functional images. Photoacoustic/ultrasound functional imaging of sO_2_ in mice brains at 750 and 850 nm produced images in which the red color represents a higher sO_2_ level and the blue color represents a lower sO_2_ level (Figure 6D,E). Two identical regions of interest (ROIs) in each image were indicated by dashed circles. The results, as shown in Figure 6D, illustrated sO_2_ in the ipsilateral cortex of eMCAO mice decreased sharply. The ratio of sO_2_ levels of MiCM‐NPs, anti‐RGMa, and uncoated‐NPs group were higher than model group. Compared anti‐RGMa and uncoated‐NPs, the sO_2_ ratio levels of MiCM‐NPs are highest. The results suggested that MiCM‐NP treatment may improve the oxygenation of the injured cortex. Moreover, the infarct area was evaluated by TTC staining (Figure 6F) seven days after treatment. The results, as shown in Figure 6G, illustrated that compared with other groups, the MiCM‐NPs group had an obvious reduction in infarct volume. Taken together, these data in Figure 6 indicated that MiCM‐NPs protected the brain from ischemia/reperfusion injury. The neuroprotection effect of MiCM‐NPs surpassed that of anti‐RGMa alone and uncoated‐NPs. The protective effect of uncoated‐NPs and anti‐RGMa alone could be attributed to distinct mechanisms. Specifically, uncoated‐NPs protected ischemic area by partially recanalize the occluded tissue, whereas anti‐RGMa alone protected the damaged tissue from ischemia/reperfusion injury. MiCM‐NPs targeted the thrombus area and recanalize the occluded vessel, which enabled more damaged tissues to be protected by anti‐RGMa.

### 2.7 Neuroprotection Mechanism of MiCM‐NPs

We explored the mechanisms of neuroprotection of MiCM‐NPs after ischemia/reperfusion injury. In our previous studies, we have confirmed that RGMa promoted BBB dysfunction,^[^ ^18^ ^]^ microglia M1 polarization,^[^ ^19^ ^]^ and reactive astrogliosis,^[^ ^17^ ^]^ while inibiting microglia M2 polarization after ischemia/reperfusion injury. Therefore, we speculated that MiCM‐NPs would protect the damaged tissue from ischemia/reperfusion injury by reducing BBB dysfunction, microglia M1 polarization and reactive astrogliosis, while promoting microglia M2 polarization after stroke as the nanoparticles release anti‐RGMa. To confirm our hypothesis, the eMCAO mice were randomly divided into four groups: Sham, eMCAO, NAb‐NPs (receiving an injection of MiCM@PLGA/Fe_3_O_4_@PFH NPs after eMCAO), and MiCM‐NPs (receiving an injection of MiCM@PLGA/anti‐RGMa/Fe_3_O_4_@PFH NPs after eMCAO).

**Figure 7:**
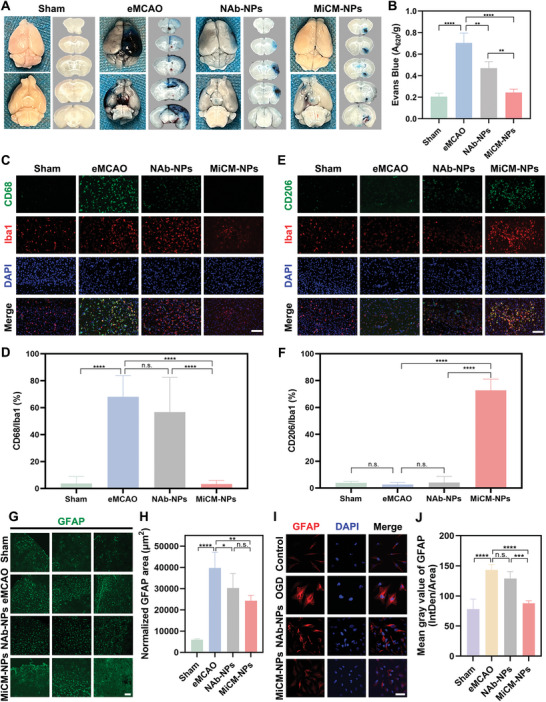
Neuroprotection mechanisms of MiCM‐NPs. Representative images A) and quantification B) of Evans blue extravasation 24 h after eMCAO in mice (n = 3). Representative images of CD68 C) and CD206 E) expression in Iba1^+^ cells in eMCAO mice at 7 days post stroke by immunofluorescence staining. Scale bar: 100 µm. D) Quantification of the ratio of CD68 in Iba1^+^ cells in each group. Scale bar: 100 µm (n = 6). F) Quantification of the ratio of CD206 in Iba1^+^ cells in each group. Scale bar: 100 µm (n = 6). Representative immunofluorescence images of GFAP expression G) and quantification H) in tissue sections of eMCAO mice at 7 days post stroke. Scale bar, 100 µm (n = 5). Representative immunofluorescence images I) and quantification J) of GFAP expression in U251MG astrocytes after OGD under different treatments. Scale bar, 100 µm (n = 5). Data show the mean ± SD. Data comparisons area made using one‐way analysis of variance (ANOVA) with Tukey's post hoc test and student's paired t‐test; *p < 0.05, **p < 0.01, ***p < 0.01, ****p < 0.0001, n.s. no significance. NAb‐NPs, MiCM@PLGA/Fe_3_O_4_@PFH NPs. MiCM‐NPs, MiCM@PLGA/anti‐RGMa/Fe_3_O_4_@PFH NPs.

Firstly, we investigated the effect of MiCM‐NPs in BBB permeability. Ischemia/reperfusion injury leads to the damage of BBB and results in brain vasogenic edema.^[^ ^35^, ^36^ ^]^ BBB damage was evaluated via quantitative Evans blue (EB) at 24 h after eMCAO. As shown in **Figure** **7**A,B, MiCM‐NPs treatment significantly reduced the extravasation of EB in eMCAO mice, suggesting that MiCM‐NPs could reduce BBB dysfunction. NAb‐NPs treatment slightly reduced the extravasation of EB, possibly due to the recanalization effect of the nanoparticles, which attenuates the area of BBB damage.

It has been widely confirmed that microglia would be activated and polarized after stroke. Some microglia polarized to pro‐inflammatory M1 phenotype releases pro‐inflammatory mediators and the others polarized to anti‐inflammatory M2 phenotype secretes anti‐inflammatory cytokines.^[^ ^37^, ^38^, ^39^ ^]^ In current study, MiCM‐NPs treatment not only significantly reduced the cellular counts of CD68^+^/Iba1^+^ M1 cells (Figure 7C,D), but also obviously increased the cellular counts of CD206^+^/Iba1^+^ M2 cells at 7 days after eMCAO in mice (Figure 7E,F). These results indicated that MiCM‐NPs could reduce microglia pro‐inflammatory M1 polarization and promote microglia anti‐inflammatory M2 polarization.

Next, we investigated the role of MiCM‐NPs in reactive astrogliosis in vivo and in vitro. In response to stroke, astrocytes convert to a reactive phenotype (so‐called reactive astrogliosis). Then the reactive astrocytes migrate to the lesion site and proliferate at the lesion margin to become the major components of the glial scar, which inhibit axon regeneration.^[^ ^40^ ^]^ The glial fibrillary acidic protein (GFAP) expression in the glial scar was detected by immunostaining in the brain at 7 days after eMCAO. Compared with eMCAO group, mice treated with MiCM‐NPs showed a reduction in GFAP immunoreactivity at 7 days following eMCAO (Figure 7G,H). The minor reduction effect of NAb‐NPs on reactive astrogliosis may also because of the recanalization effect of the nanoparticles, which decreases injured areas. In in vitro study, we establish an OGD model in U251MG astrocytes. After OGD, the astrocytes received NAb‐NPs or MiCM‐NPs treatment, followed by an external LIFU treatment. Astrocytes induced by OGD showed a high GFAP expression with a hypertrophic morphology, while MiCM‐NPs‐induced astrocytes displayed reduced GFAP immunoreactivity and were less hypertrophic (Figure 7I,J). Collectively, these results showed MiCM‐NPs could inhibit reactive astrogliosis after stroke.

### 2.8 Safety of MiCM‐NPs

**Figure 8:**
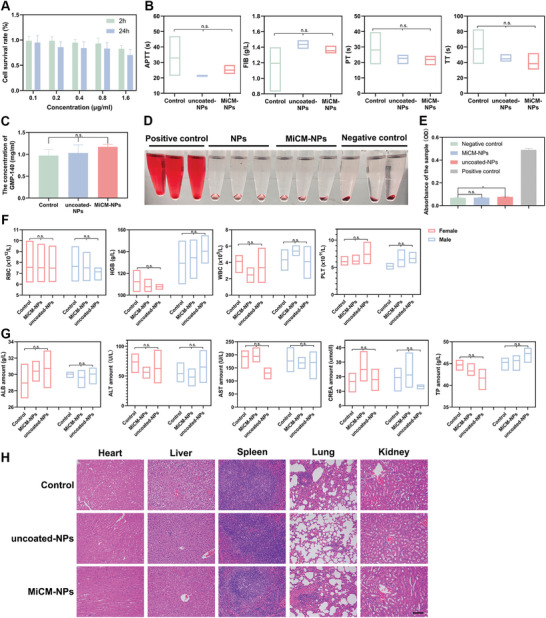
Safety evaluation of MiCM‐NPs. A) Relative viabilities of bEnd.3 cells received different concentration of MiCM‐NPs treatment at 2 h and 24 h (n = 5). Coagulation B) and GMP‐140 C) in plasma in normal mice received normal saline (Control), PLGA/anti‐RGMa/Fe_3_O_4_@PFH (uncoated‐NPs), and MiCM@PLGA/anti‐RGMa/Fe_3_O_4_@PFH (MiCM‐NPs) treatment (n = 3). Images D) and erythrocyte hemolysis percentages E) of 2% red blood cell (RBC) PBS solutions treated with PLGA/anti‐RGMa/Fe_3_O_4_@PFH (uncoated‐NPs) or MiCM@PLGA/anti‐RGMa/Fe_3_O_4_@PFH (MiCM‐NPs). PBS and Triton X‐100 were used as negative and positive control, respectively (n = 3). Levels of hematological parameters F) and biochemical markers G) relevant to hepsatic and kidney functions in serum (n = 3). H) H&E staining of major organ of mice in Control, PLGA/anti‐RGMa/Fe_3_O_4_@PFH (uncoated‐NPs) and MiCM@PLGA/anti‐RGMa/Fe_3_O_4_@PFH (MiCM‐NPs) group. Scale bar, 100 µm. Data show the mean ± SD. Data comparisons are made using one‐way analysis of variance (ANOVA) with Tukey's post hoc test; *p < 0.05; n.s. no significance.

The toxicity of nanomaterials for biomedical application is always a concern. We evaluated the cell viability of MiCM‐NPs. MiCM‐NPs were cultivated with bEnd.3 cells for 2 or 24 h. Results showed that MiCM‐NPs did not cause damage to the endothelial cells (**Figure** **8**A). Moreover, due to the possible activation of the coagulation cascade by MiCM‐NPs and the effect of ultrasound on hemodynamics, we investigated the effect of NP treatment on the coagulation system in mice. We injected 2 mg mL^−1^ of NPs into mice, applied excitation with LIFU and positioned in the magnetic field, and extracted mouse plasma for coagulation examination. Experimental data showed no significant difference among the MiCM‐NPs group, uncoated‐NPs group, and control group (Figure 8B). GMP‐140 is a platelet α‐granule membrane protein that can be synthesized by vascular endothelial cells and used as a marker of early endothelial activation time to assess the effect of platelet activation in vitro and in vivo.^[^ ^41^, ^42^ ^]^ Enzyme‐linked immunosorbent assay (ELISA) was used to detect the expression of GMP‐140 antibodies in blood to determine whether the fabricated material had the potential to trigger secondary thrombosis (Figure 8C). No visible differences in GMP‐140 concentrations were observed among the MiCM‐NPs group, uncoated‐NPs group, and control group, indicating that the injection of the materials did not cause a coagulation response in the organism. Moreover, nanomedicines were administrated by intravenous injection since the blood compatibility of nanomedicines is an important safety index. Herein, the hemolysis test was carried out to evaluate the blood biocompatibility of NPs. Same with the negative control group, the dilute blood incubated with uncoated‐NPs and MiCM‐NPs presented as a clear solution after centrifugation (Figure 8D,E), indicating that MiCM‐NPs were safe application in vivo. We also monitored the blood routine and blood biochemistry of MiCM‐NPs. As expected, the hematological indexes in the MiCM‐NPs and uncoated‐NPs groups had no obvious variations (Figure 8F,G). In addition, the H&E staining results of tissue sections showed no significant abnormalities, indicating that MiCM‐NPs did not cause any organ toxicity (Figure 8H). The combination of these results revealed the high therapeutic biosafety of MiCM‐NPs.

## 3 Conclusion

In this study, we developed an artificial microglia nanoplatform loaded with anti‐RGMa in acoustic/magnetic field for recanalization and neuroprotection in acute ischemic stroke. Following intravenous delivery, the nanoplatform targeted the ischemia‐damaged endothelial cells of the BBB in the thrombus area of stroke due to the microglia membrane coating. Fe_3_O_4_ and anti‐RGMa were released into the thrombus area after external LIFU exposure. The occluded vessel that caused the stroke was then recanalized by disruption of thrombus via LIFU‐induced “liquid‐to‐gas” phase transition and cavitation of PFH as well as Fe_3_O_4_ movements and rotations induced by an external magnetic field. Finally, anti‐RGMa in the ischemic area protected against ischemia/reperfusion injury. Thus, our nanoplatform promoted functional recovery and minimized the infarct volume. Additionally, the NP enabled the visualization of the thrombus by ultrasound/photoacoustic imaging when the clot was in an extracranial artery. This study provided an ischemic stroke treatment strategy that combined recanalization with neuroprotection.

## 4 Experimental Section

### Materials

PLGA (75% propyl cross‐ester and 25% ethyl cross‐ester, molecular weight 8000, end group = COOH) was purchased from Jinan Daigang Biomaterials Ltd. (Shandong, China). PFH was from J&K Scientific Ltd. Iron oxide NPs (Fe_3_O_4_, 10 nm) containing oleic acid were purchased from Ocean Nano Technology Co. RGMA antibody was obtained from Hangzhou Hua'an Biotechnology Co. Poly(vinyl alcohol) (PVA) with a mean molecular weight of 30 000–70 000, TTC, and 4′,6‐diamidino2‐phenylindole (DAPI) were purchased from Sigma‐Aldrich (St. Louis, Missouri, USA). Isopropyl alcohol was purchased from Shanghai Maclean Biochemical Technology Co. DiO, DiI, Coomassie Blue Fast Staining Solution, Membrane and Cytosol Protein Extraction Kit and CCK‐8 were purchased from Beyotime Biotechnology (Jiangsu, China). 1,1‐dioctadecyl‐3,3,3,3‐tetramethylindotricarbocyanine (DIR) was from AAT Bioquest (California, USA). Agarose was purchased from Invitrogen (Carlsbad, CA, USA). Helmholtz coils were purchased from Hunan Paisheng Science and Technology Co. Ltd. (Hunan, China). TMEM119 (27585‐1‐AP, proteintech), Cx3cr1 (66915‐1‐Ig, proteintech), GFAP (#3670, Cell Signaling Technology), CD206 (24595S, Cell Signaling Technology), Iba1 (OB‐MMS039‐02, oasis biofarm), CD68 (OB‐PRB044‐02, oasis biofarm).

### Preparation of MiCM‐NPs

MiCM‐NPs were prepared using a warter‐in‐oil (W/O/W) method.^[^ ^8^, ^43^, ^44^, ^45^ ^]^ First, PLGA (25 mg) and Fe_3_O_4_ (25 mg mL^−1^, 100 µL) were fully dissolved in dichloromethane (2 mL) (oil phase, O); then, PFH (0.2 mL) and anti‐RGMa (9.46 mg) (inner aqueous phase, W1) were added. The mixture was oscillated at 52 W energy for 6 min (5 s: on, 5 s: off) using an acoustic vibration ultrasonic oscillation instrument (SONICS & MATERIALS, Inc., USA), producing a brown emulsion. A PVA solution (2%, 4 mL) was then added to the above emulsion and emulsify for 6 min at 40 W energy (5s: on, 5s: off) using the ultrasonic oscillation instrument. All operations were conducted in an ice bath. Then 2‐propanol (2%, 5 mL) was added into the solution and the sample was left overnight at 4 °C until the dichloromethane evaporated and the surfaces of the NPs solidified. Subsequently, the solution was centrifuged at 7 500 rpm for 5 min, the supernatant was discarded, and the precipitate was washed with deionized water. The process of washing and centrifugation was repeated three times. Finally, the uncoated‐NPs were collected and stored at 4 °C for further use. Microglia membrane was extracted from LPS‐activated BV‐2 microglia: Briefly, LPS‐activated BV‐2 cell were grown to 80% confluence in multiple T75 culture flask and harvested, followed by washing in 1X PBS three times by centrifuging at 1 000 rad for 5 min. The purified cells underwent three cycles of freezing and thawing from −80 °C to 37 °C, followed by enucleation using a hand‐held Dounce homogenizer (WIGGENS, Germany). After centrifugation at 700 × g for 5 min at 4 °C, the supernatant was collected. Then the supernatant was centrifuged at 15 000 × g for 30 min and the precipitate obtained after centrifugation is the microglia membrane. The microglia membrane was added to the PLGA/anti‐RGMa/Fe_3_O_4_@PFH suspension at a PLGA‐to‐membrane protein weight ratio of 1:1. The mixture was sonicated for 1 min (40 W energy, 5 s: on, 5 s: off) in an ice water bath. Finally, the MiCM‐NPs were collected and stored at 4 °C for further use. For DiO/DiI/DiR‐labeling experiments, the DiO, DiI, or DiR (100 µM, 20 µL) was dissolved in PLGA.

### Characterization of MiCM‐NPs

After the appropriate MiCM‐NPs were dissolved in double‐distilled water, the internal structures were observed using a TEM (JEM 2100, JEOL Ltd., Tokyo, Japan). To observe the distribution of elements in MiCM‐NPs, the authors acquired high‐resolution TEM images (HRTEM), and elemental mapping using a different TEM instrument (FEI Tecnai G20 F30, FEI Co., USA). The sizes and zeta potentials of nanoparticles were determined at 25 °C using a laser particle size analyzer (Zetasizer Nano ZS90, Malvern Instruments Ltd., Worcs, UK). The attachment of the DiI‐labeled MiCM film to the DiO‐labeled uncoated‐NPs was confirmed using a microscope (A1+R, Nikon, Japan). Saturation magnetization values of MiCM‐NPs were measured by a hysteresis loop test (LakeShore 7404, American). The whole protein analysis of MiCM, NAb‐NPs, PLGA/Fe_3_O_4_@PFH, anti‐RGMa, and uncoated‐NPs was performed by SDS‐PAGE. All the samples were centrifuged at 14 000 rpm for 10 min, prepared at a protein (600 mg mL^−1^) mixed with 4X loading buffer, heated at 95 °C for 10 min, and the protein (10 µL) loaded onto 10% bis‐tris protein gels. Staining was performed with Coomassie brilliant blue. For western‐blot analysis, the protein was transferred onto a polyvinylidene difluoride membrane and treated with primary antibodies against TMEM119 and Cx3cr1 as well as HRP conjugated anti‐mouse/rabbit IgG secondary antibody.

### Encapsulation Efficiency, Drug Loading, and Drug Release of MiCM‐NPs

FITC‐labeled anti‐RGMA was used to determine the encapsulation efficiency (EE and drug loading (DL), using the following equation:

(1) $$\begin{array}{c} \mathrm{Encapsulation}\;\mathrm{Efficiency}\;\left(EE\right)=\frac{mass\;of\;the\;drug\;in\;the\;NPs}{total\;initial\;mass\;of\;the\;drug}\times \;100\% \end{array}$$

(2) $$\mathrm{Drug}\;\mathrm{Loading}\;\left(\mathrm{DL}\right)=\frac{mass\;of\;the\;drug\;in\;NPs}{mass\;of\;NPs}\times 100\%$$

The parameters of LIFU were adjusted to pulse mode (2 s: on, 2 s: off). The MiCM‐NPs with FITC‐labeled anti‐RGMA was loaded into a 5 mL EP tube, placed at the center of the LIFU probe, and irradiated with different powers (1, 2, 3, 4 W cm^−2^) for 5 min. Centrifugation was utilized to separate the free FITC‐labeled anti‐RGMa and MiCM‐NPs, at the speed of 14 000 rpm for 20 min. Drug release efficiency was analyzed by measuring the absorbance at 488 nm.

### Animals and Cell Lines

Animals for the experiments were purchased from the Laboratory Animal Center of Chongqing Medical University.All animal experiments conformed to the Animal Ethics Committee Guidelines of Chongqing Medical University, and the protocols were approved by the Animal Protection Committee of Chongqing Medical University (Ethics No. IACUC‐CQMU‐2023‐0144). BV‐2 cell, U251MG cell and bEnd.3 cell were obtained from Chongqing Key Laboratory of Major Neurological and Mental Disorders.

### Preparation of Blood Clots

Femoral arterial cannulation was performed on a donor rat. Femoral artery blood was transferred directly into a PE‐50 tube. The tube was stored at room temperature for 2 h to clot blood and then at 4 °C for 22 h.

### LCCA Embolization Model

Sprague‐Dawley (SD) rats (male; 4–6 weeks old; 100 to 150 g body weight) were obtained from the Laboratory Animal Center of Chongqing Medical University for in vivo experiments. The rats were anesthetized (200 mg mL^−1^ tribromoethanol), and the left common carotid artery of each rat was exposed by removing the skin and fascia. Then, vascular thrombosis was induced by placing a filter paper strip saturated with FeCl_3_ (10%) over the vessels. After 10 min, the filter paper was removed and rinsed with saline. Throughout the operation, body temperature was maintained at 37 °C by placing the rats under a heat lamp.

### eMCAO

C57 mice (male; 6–8 weeks old; about 20 g body weight) were obtained from the Laboratory Animal Center of Chongqing Medical University for in vivo experiments. Mice and clots were subjected to the procedures reported in a past study.^[^ ^46^ ^]^ After anesthetization, the right common carotid artery (CCA), the right external carotid artery (ECA), and the right internal carotid artery (ICA) were exposed. A modified PE‐8 catheter containing a blood clot was inserted into the incision and through the right ECA into the lumen of the right ICA until it encountered the origin of the right middle cerebral artery (MCA). Using the modified PE‐8 catheter and a syringe, the blood clot and saline (100 µL) were injected over 3 min, after which the catheter was withdrawn. Throughout the operation, body temperature was maintained at 37 °C by placing the mice under a heat lamp.

### Motility Characterization and Thrombolysis Process of Fe_3_O_4_ Under Magnetic Field in Vitro

For the dynamic experiment in a flowing environment, a peristaltic pump was utilized to produce a liquid circulating environment with a flow rate of 3 mL min^−1^. Then, Fe_3_O_4_ (25 mg mL^−1^, 20 µL) was placed in a PE‐tube filled with normal saline, and the magnet was placed next to the PE‐tube. A Helmholtz coil system was constructed, and each coil was controlled independently by a current controller. The generated magnetic field was up to 40 mT and was used to provide magnetic guidance. To test the thrombolysis process of Fe_3_O_4_, we performed in vitro experience. A clot was placed in a flow chamber consisting of a transparent PE‐tube, and PBS was circulated through the chamber at the rate of 3 mL min^−1^ by a peristaltic pump (Figure S3, Supporting Information). Subsequently, Fe_3_O_4_ was infused into the PBS from the bottom of the tube using a syringe needle. The clot was exposed to a rotating magnetic field (30 mT, 4 Hz) and treated for 60 min. Finally, all the clots were collected and washed by PBS for H&E staining to evaluate the thrombolysis efficacy under a magnetic field in vitro.

### Multimodal Imaging of the MiCM‐NPs in Vitro and in Vivo

To study the imaging properties of the MiCM‐NPs in vitro, we made a 3% agarose gel cast of a 200 µL conical pipette tip. Next, MiCM‐NPs (1 mg mL^−1^, 100 µL) was added to an agarose gel, and double‐distilled water was used as control. The agarose gel models were irradiated with LIFU (LMSC051ACA; Institute of Ultrasound Imaging, Chongqing Medical Sciences, Chongqing, China) for 1, 2, 3, and 4 min at an acoustic power density of 1 W cm^−2^ to 4 W cm^−2^. Ultrasonic images in B‐mode and CEUS mode were collected after ultrasonic irradiation. Aqueous solutions of MiCM‐NPs at different concentrations of Fe_3_O_4_ content (0.625, 1.25, 2.5, 5, 10 mg mL^−1^) were added to this agarose gel model to acquire photoacoustic images.

The imaging properties in vivo: The LCCA model rats (n = 3) were intravenously injected with MiCM‐NPs (5 mg mL^−1^, 0.5 mL), and a magnet was used at the thrombus site. After 60 min, rats were irradiated with LIFU at 3 W cm^−2^ for 3 min. Imaging was performed at a series of time points and the images of B‐mode, CEUS, and photoacoustic‐mode were collected.

Grayscale quantitative analysis software (Institute of Ultrasound Imaging, Chongqing Medical University) was used to calculate the average echo value of the ultrasonic images in B‐mode and CEUS. A Vevo LAZR photoacoustic imaging system (VisualSonics Inc., Toronto, Canada) with a laser at an excitation wavelength of 705 nm was used to acquire photoacoustic images. Image analysis was carried out using the same Vevo LAZR photoacoustic imaging system.

### In Vitro Targeting of MiCM‐NPs

The bEnd.3 cells were cultured in 10% FBS (catalog no. C0235; Gibco, Australia) and 1% penicillin/streptomycin (catalog no. C0222; Beyotime, China) in DMEM (catalog no. 88 287; Gibco MA). Cells were cultured at 37 °C and 5% CO_2_ in a humidified environment. A total of 1 × 10^6^ bEnd.3 cells were cultured in and 6‐well plates, treated with oxygen‐glucose deprivation for 6 h, and then treated with DiO‐labeled uncoated‐NPs, MiCM‐NPs, and MaCM‐NPs (0.25 mg mL^−1^, 50 mL) for 3, 6, and 9 h. The cells were washed with precooled 1X PBS three times, stained with DAPI, observed with an inverted fluorescent microscope, and analyzed by flow cytometry.

### In Vivo Targeting of MiCM‐NPs

The eMCAO mice (n = 3) were divided into three groups (uncoated‐NPs group, MaCM‐NPs group, and MiCM‐NPs group). The eMCAO mice were intravenously administered DiR‐labeled uncoated‐NPs, MaCM‐NPs, or MiCM‐NPs (10mg k^−1^g) 1 h after eMCAO through tail vein. The mice in the three groups were sacrificed by injecting an overdose of pentobarbital (160 mg kg^−1^) at 120 min postinjection, and the brains were harvested for ex vivo fluorescence imaging. The brain of MiCM‐NPs group was also collected for immunofluorescence staining. Briefly, the brain was fixed in 4% paraformaldehyde for 1 day, and brain sections were counterstained with DAPI for 10 min and protected by antifade mounting medium. Images were captured using a confocal laser scanning microscope (Dragonfly 200, Andor, UK) and analyzed by ImageJ software.

### Half‐life Measurement of MiCM‐NPs

C57 mice were divided into two groups (uncoated‐NPs group and MiCM‐NPs group). Next, DiR‐labeled uncoated‐NPs or MiCM‐NPs (2 mg mL^−1^, 200 µL) were injected intravenously into the mice, and blood samples (300 µL) were collected from inner eyes at preset times (1, 2, 6, 12, 24, and 48 h). After centrifugation at 12 500 rpm for 5 min, plasma was obtained and the fluorescence signals were detected by a full‐band multifunctional microplate reader.

### Recanalization and Safety Evaluation of LIFU and Magnetic Responsive MiCM‐NPs in Vivo

After LCCA embolization was modeled in rats, MiCM‐NP (5 mg mL^−1^, 0.5 mL) was given by LIFU excitation at 3 W cm^−2^ for 6 min (2 s: on, 2 s: off) and the thrombus site was placed in the Helmholtz coil system for 15 min; this process was repeated three times. The thrombolytic effect in LCCA embolization was measured by pulsed wave Doppler and evaluated by PSV of LCCA. The LCCA were simultaneously visualized by H&E staining, Masson staining, elastic fiber staining, and reticular fiber staining to detect vascular injury.

### The Neuroprotection Evaluation of MiCM‐NPs in Vivo—The Behavior Test

The mice were trained before the experiment according to the mNss scoring standard and the instructions of the experimental equipment for cylinder test and rotating test.^[^ ^47^ ^]^ Successfully trained mice were selected for the experiment after 3 days of training. Three mice were selected as the sham group and 15 mice were selected as eMCAO mice, which were randomly assigned to the model, IgG, anti‐RGMa, uncoated‐NPs, and MiCM‐NPs groups (n = 3 per group). The eMCAO mice were intravenously administered normal saline, IgG, uncoated‐NPs, anti‐RGMa, or MiCM‐NPs (10mg k^−1^g). 60 min after eMCAO mice modeled, they were subjected to LIFU excitation at 2 W cm^−2^ for 3 min (2 s: on, 2 s: off) and then the thrombus site was placed in the Helmholtz coil system for 5 min; this process was repeated for a total of two times. The mNss scores and behavioral test were performed on days 1, 3, and 7 after treatment.

### Oxygen Saturation (sO_2_) and TTC

At 7 days after treatment, mice were anesthetized using tribromoethanol (200 mg mL^−1^) and secured onto the imaging platform underneath the PAI transducer. Non‐invasive photoacoustic imaging of sO_2_ was measured by the Vevo LAZR photoacoustic imaging system with the laser at 750 and 850 nm. All the slices were divided into left and right parts. Two identical ROIs (ROI_left_ and ROI_right_) in each image were indicated by dashed circles. The relative photoacoustic imaging signal of sO_2_ was analyzed by the equation:

(3) $$sO_{2}\;\mathrm{Avr}\;\left(\%\right)=\frac{\mathrm{RO}I_{\mathrm{left}}}{\mathrm{RO}I_{\mathrm{right}}}\times 100\%$$

Then the mice were sacrificed and subjected to transcranial perfusion with normal saline. The brains were harvested, frozen at −20 °C, and dissected into 2 mm thick sections. The fresh brain slices were stained with TTC solution for 30 min at 37 °C and immersed in 4% paraformaldehyde for 24 h. The infarct areas (white parts) in the images were quantified with ImageJ software.

### Immunofluorescence Staining in eMCAO Mice

At 7 days after treatment, the frozen brain sections, after antigen retrieval using sodium citrate buffer, were permeabilized using 0.1% Triton X‐100 and blocked with 10% normal donkey serum. Then the sections were incubated overnight at 4 °C with specific primary antibodies as follows: Iba‐1 antibody (1:500), CD206 antibody (1:300), CD68 antibody (1:500), GFAP antibody (1:300). Secondary antibodies including Alexa Fluor 488 and Alexa Fluor 594 were utilized with 1:1000 dilution and stained with DAPI before observation under CLSM (Dragonfly 200, Andor, UK). The fluorescence intensity or positive cells were quantified with ImageJ for further analysis.

### Immunofluorescence Staining in BV‐2 Cell and U251MG Cell

BV‐2 microglia and U251MG astrocytes were cultured in 10% FBS and 1% penicillin/streptomycin in DMEM. Cells were cultured at 37 °C and 5% CO_2_ in a humidified environment. The cells were cultured in confocal dish plates, treated with OGD for 6 h (U251MG).^[^ ^48^ ^]^ Then treated with NAb‐NPs or MiCM‐NPs (0.25 mg mL^−1^, 50 mL). After LIFU (2 W cm^−2^,2 min) activation, the cell was co‐cultured for 24 h. For cell staining, cells were fixed with 4% fixative solution for 30 min at 37 °C. After washing with PBS, cells were permeabilized using 0.3% Triton X‐100 and blocked by 10% normal donkey serum for 1 h at 37 °C. Then incubated overnight at 4 °C with primary antibodies including Iba‐1 antibody (1:500), CD206 antibody (1:300), CD68 antibody (1:500), GFAP (1:300). Cells were washed and incubated for 1 h at 37 °C with secondary antibodies conjugated with Alexa Fluor 488 or 594. Sections or cells were stained for DAPI to visualize nuclei. Images were captured using confocal laser scanning microscope (A1+R, Nikon, Japan) and analyzed by Image J software (National Institutes of Health).

### Evans Blue Dye Leakage Assay

Evans blue dye (2%) was injected through the tail vein of eMCAO mice after one day. After 2 h, the mice were administered transcranial perfusion with 50 ml of ice‐cold PBS, to wash out the intravascular dye. Then the brains were cut into sections and photographed to observe the Evans blue ‐stained area. The concentration of Evans blue was measured by spectrophotometry, and the results were expressed in A_620_/g of brain tissue.^[^ ^35^ ^]^

### Erythrocyte Hemolysis Assay

Red blood cells (RBCs) were gently extracted from fresh blood to prepare 2% RBC in saline. Then the RBC solution was incubated with PBS, 1% Triton X‐100, uncoated‐NPs, and MiCM‐NPs for 3 h. Digital pictures were taken at 3 h. After centrifugation, hemoglobin in the supernatant was detected by a microplate reader at 570 nm.

### Toxicity and Safety Evaluation of MiCM‐NPs

The bEnd.3 cells were placed in 96‐well plates (2 × 10^4^ cells well^−1^) and incubated until they reached 60% confluence. MiCM‐NPs were added at various concentrations (0.1, 0.2, 0.4, 0.8, and 1.6 mg mL^−1^) for 2 and 24 h, and the medium was discarded at a specific point in time. The medium containing CCK8 reagent was added, and the mixture was incubated at 37 °C for 2 h. Then, the absorbance of the above medium was determined with a microplate reader (Bio‐Tek Instrument Inc., USA) at a wavelength of 450 nm.

C57 mice were used for in vivo toxicity analysis. MiCM‐NPs (2 mg mL^−1^, 200 µL) and saline were injected into the tail vein. Fresh citrated blood was collected and spun for 10 min at 3 000 rpm min^−1^ at room temperature after treatment. The plasma was collected and sent to the clinical laboratory to determine the AP, APTT, TT, and FIB. The contents of GMP‐140 in the samples were determined with the ELISA kit according to the protocol. Furthermore, serum was obtained to measure ALT, ALB, AST CREA, and TP levels as indicators of renal and hepatic function. Whole blood samples were also obtained for routine blood examination. The heart, liver, spleen, lungs, and kidneys were harvested for pathological H&E staining for 24 h.

### Statistical Analysis

SPSS 22.0 (Chicago, Illinois, USA) and GraphPad Prism 7.0 (San Diego, CA, USA) were used for statistical analysis. All data were expressed as the mean ± standard deviation of at least three independent tests. The *t* test was used to compare the two groups. Differences between groups were analyzed using one‐way ANOVA when more than two groups were compared, and comparisons between groups were performed using post‐hoc *t* tests with Bonferroni correction. Differences were considered statistically significant at *p* < 0.05.

## Conflict of Interest

The authors declare no conflict of interest.

## Supporting information



Supporting Information

Supplemental Movie 1

Supplemental Movie 2

## Data Availability

The data that support the findings of this study are available from the corresponding author upon reasonable request.
