# Supplementary material for: Artificial Microglia Nanoplatform Loaded With Anti‐RGMa in Acoustic/Magnetic Feld for Recanalization and Neuroprotection in Acute Ischemic Stroke
Source: Adv Sci (Weinh). 2024 Oct 30;11(48):2410529. doi: 10.1002/advs.202410529 (PMC11672321; doi:10.1002/advs.202410529)
Supplement: Supplementary file 1 — Supporting Information [file ADVS-11-2410529-s001.docx]

Supporting Information

Artificial Microglia Nanoplatform Loaded with Anti-RGMa in Acoustic/Magnetic Field for Recanalization and Neuroprotection in Acute Ischemic Stroke

Ruiqi Cheng,^†^ Xiaoqin Luo,^†^ Xiaohui Wu, ^†^ Zijie Wang, Ziqun Chen, Shaoru Zhang, Hongmei Xiao, Jiaju Zhong, Rongrong Zhang,* Yang Cao,* Xinyue Qin*


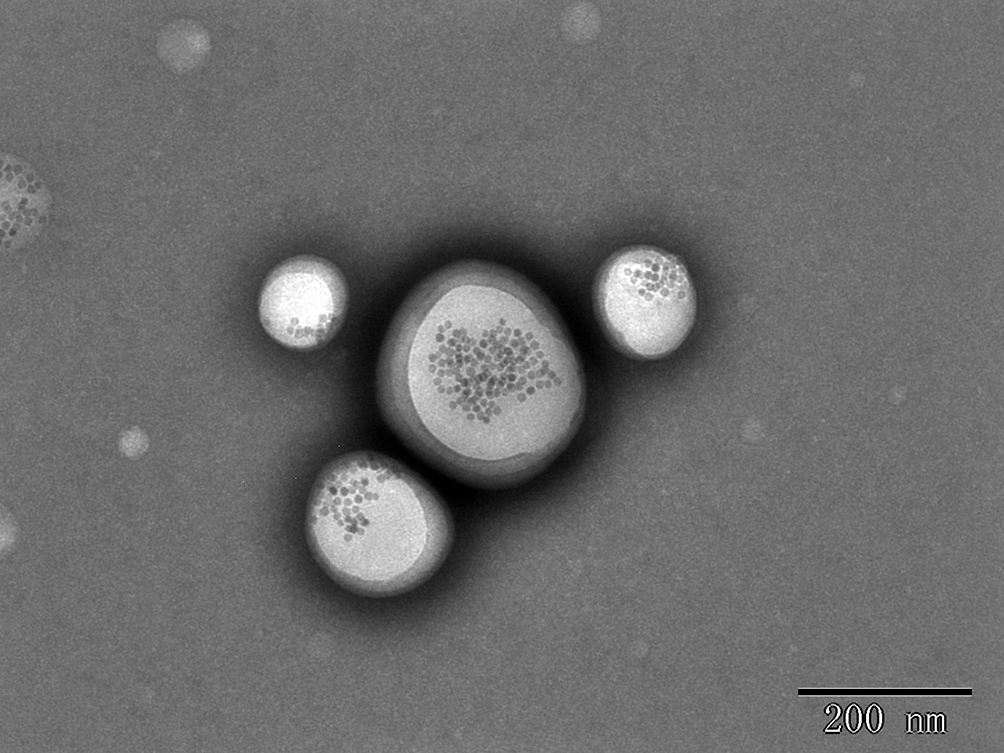


**Figure S1.** Representative TEM image of MiCM-NPs. Scale bar, 200 nm.


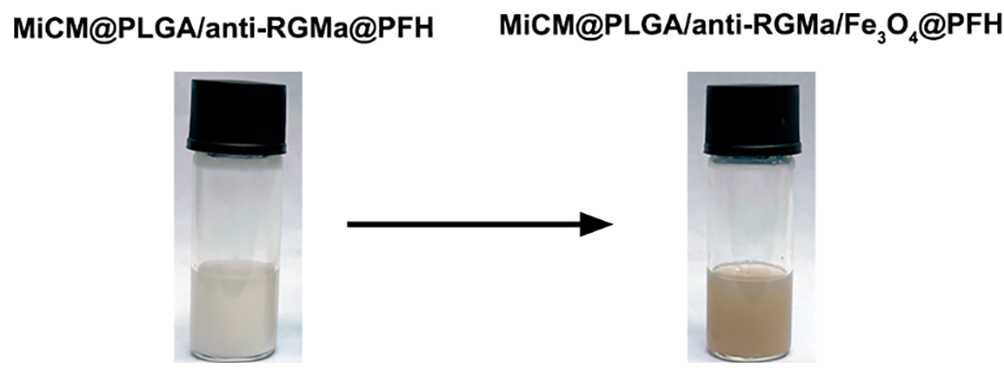


**Figure S2.** Photograph images of MiCM@PLGA/anti-RGMa@PFH (left) and MiCM@PLGA/anti-RGMa/Fe_3_O_4_@PFH (right).


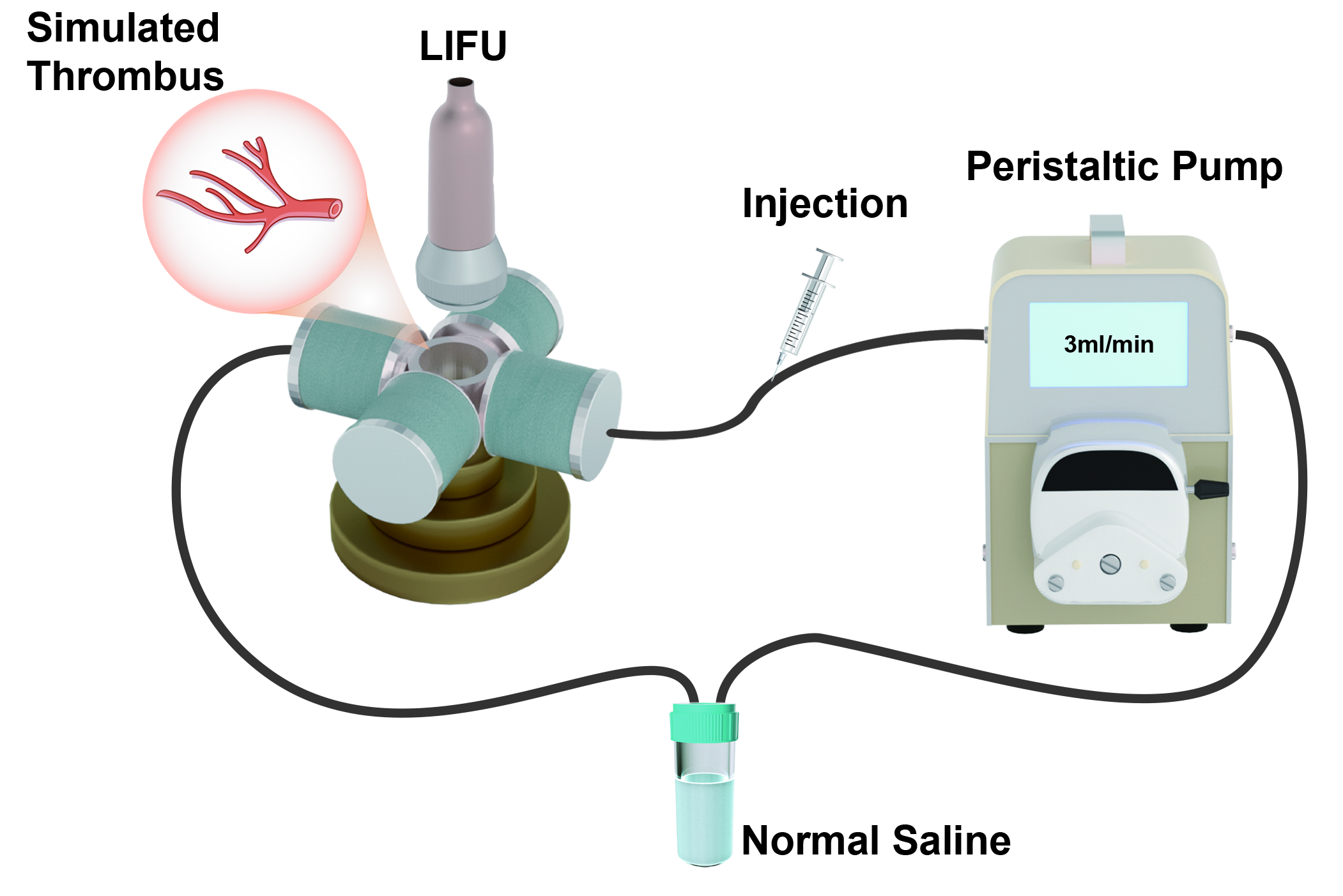


**Figure S3.** Model diagram of LIFU and magnetic combination in vitro.


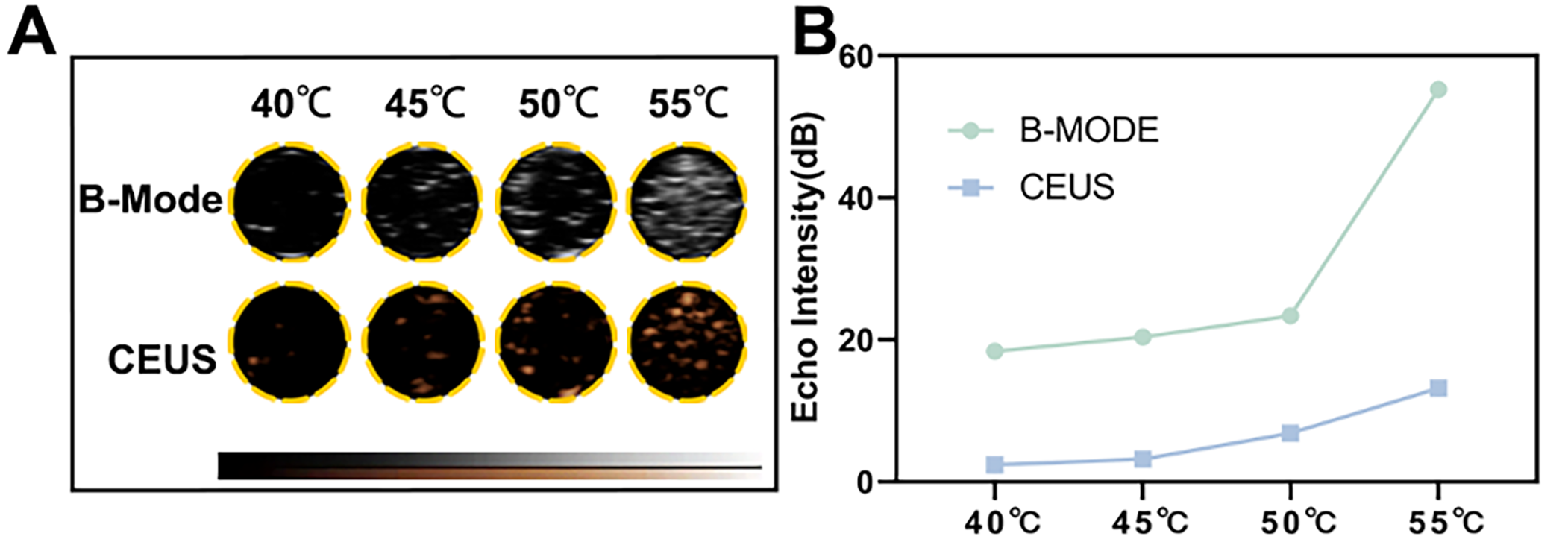


**Figure S4**. (**A**) B-mode and CEUS images of MiCM-NPs from 40℃ to 55℃ (with 5℃ intervals). (**B**) Echo intensity values of B-mode (green) and CEUS (blue) from 40℃ to 55℃ (with 5℃ intervals).


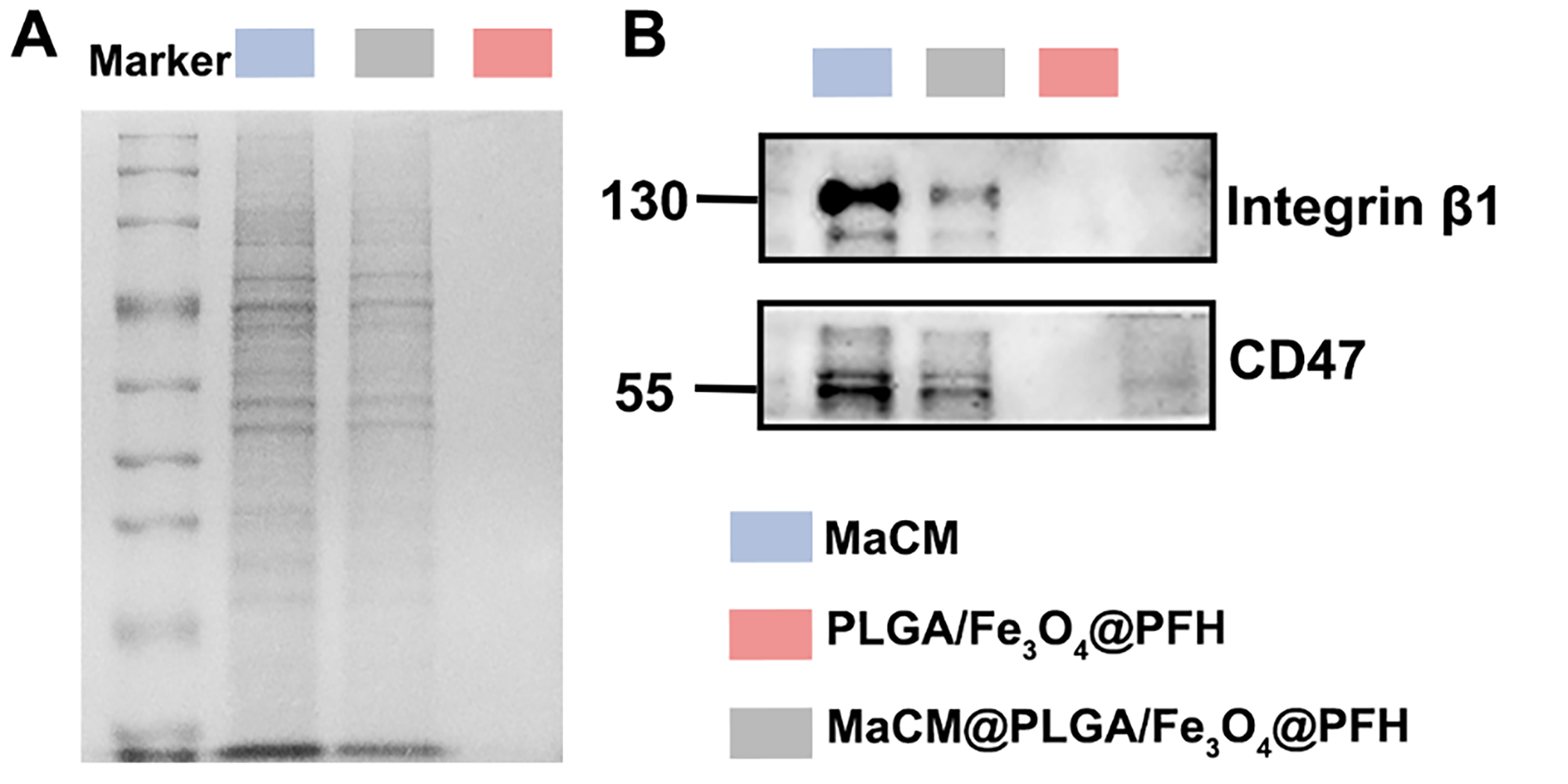


**Figure S5.** (**A**) SDS-PAGE analysis of proteins of MaCM, MaCM@PLGA/Fe_3_O_4_@PFH, and PLGA/Fe_3_O_4_@PFH. (**B**) Western-blot analysis of MaCM, MaCM@PLGA/Fe_3_O_4_@PFH, and PLGA/Fe_3_O_4_@PFH for its surface marker protein (Integrin β1 and CD47). MaCM, macrophage membrane.


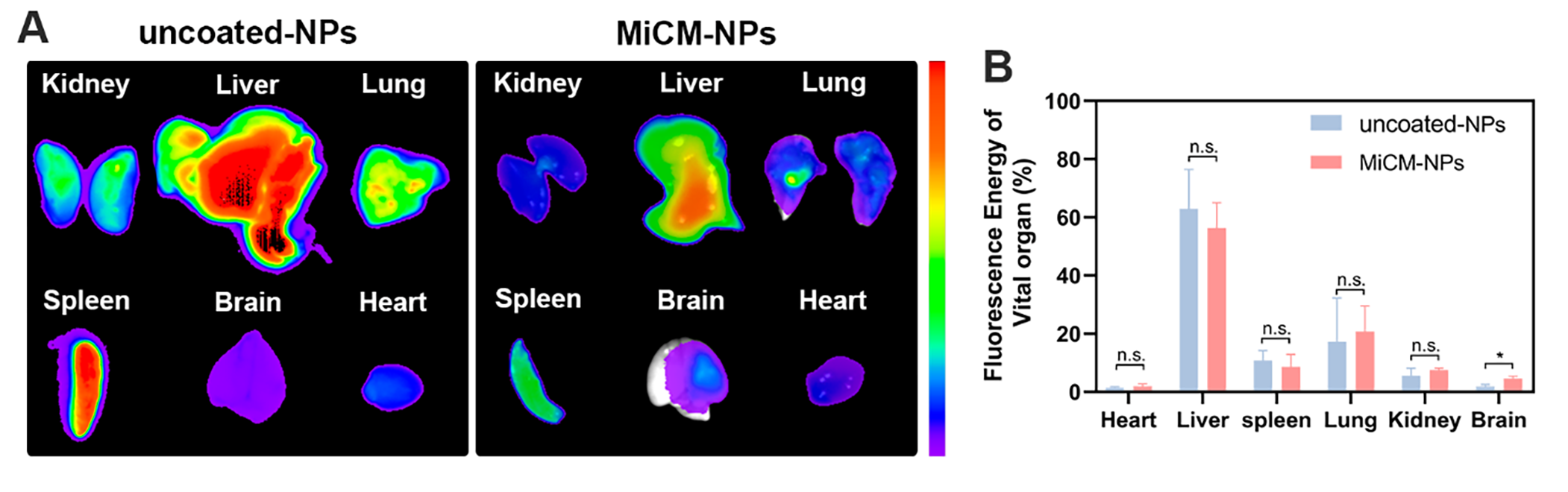


**Figure S6.** Images (**A**) and quantification (**B**) of the distribution of PLGA/anti-RGMa/Fe_3_O_4_@PFH (uncoated-NPs) and MiCM@PLGA/anti-RGMa/Fe_3_O_4_@PFH (MiCM-NPs) in the vital organs of eMCAO mice at 6 h post tail vein injection of DiR labeled uncoated-NPs and MiCM-NPs. Data show the mean ± SD. Data comparisons are made using student’s paired t-test; **P* < 0.05.


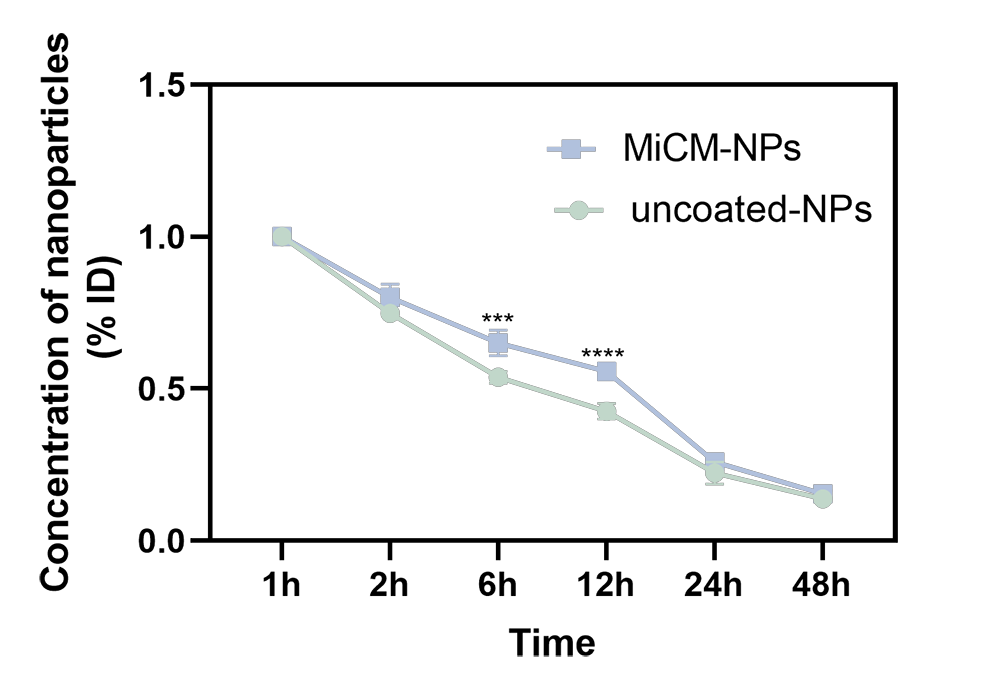


**Figure S7.** Blood retention of DiR-labeled PLGA/anti-RGMa/Fe_3_O_4_@PFH (uncoated- NPs) and MiCM@PLGA/anti-RGMa/Fe_3_O_4_@PFH (MiCM-NPs) (dosage of 200 μL of 2 mg mL^-1^) in C57 mice after single intravenous injection to evaluate the prolong circulation ability of MiCM-NPs. Values represent mean ± S.D., ***p < 0.001, ****p < 0.0001.


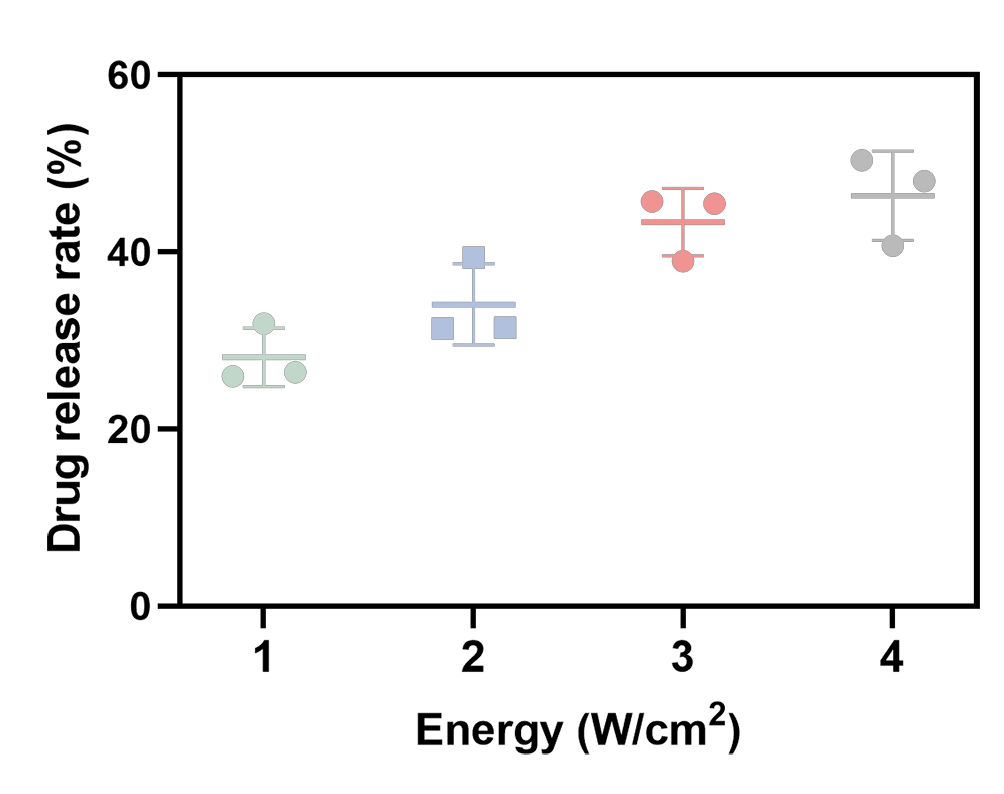


**Figure S8.** Comparative drug release characteristics of MiCM-NPs in PBS after LIFU irradiation with different power for 3 min.
